# Bridging Echinocandin With Azole Antifungal Therapy on Prevention of Invasive Candidiasis Post–Lung Transplantation

**DOI:** 10.1093/ofid/ofae525

**Published:** 2024-09-11

**Authors:** Taylor Pasley, Christopher Baladad, Kathryn DeSear, Solmaz Karimi, Eric Rubido, Guy El Helou, Maureen Converse

**Affiliations:** Department of Pharmacy, Houston Methodist Hospital, Houston, Texas, USA; Department of Pharmacy, Alvarado Hospital Medical Center, San Diego, California, USA; Department of Pharmacy, University of Florida Health Shands Hospital, Gainesville, Florida, USA; Department of Pharmacy, The Ohio State University Wexner Medical Center, Columbus, Ohio, USA; Department of Pharmacy, Houston Methodist Hospital, Houston, Texas, USA; Division of Infectious Disease and Global Medicine, Department of Medicine, University of Florida, Gainesville, Florida, USA; Department of Pharmacy, Cleveland Clinic, Cleveland, Ohio, USA

**Keywords:** antifungal prophylaxis, antimicrobial stewardship, lung transplant

## Abstract

**Background:**

Invasive candidiasis (IC) is a significant factor for lung transplant recipient (LTR) mortality, especially in the immediate postoperative phase. Receipt of antifungal prophylaxis has demonstrated lower all-cause mortality.

**Methods:**

This was a single-center, retrospective cohort study of LTRs between August 2017 and August 2020. Included patients were adult LTRs with positive *Candida* cultures preoperatively (donor or recipient) or within 6 weeks postoperatively. Patients were divided into 2 cohorts—bridged and unbridged. The bridged cohort received micafungin in the postoperative period until therapeutic azole concentrations were achieved or up to 2 weeks, whichever was sooner. The primary outcome was a composite of proven or probable invasive candidiasis.

**Results:**

A total of 117 patients were included in the study, with 68 in the unbridged cohort and 49 in the bridged cohort. There were more cases of IC in the bridged cohort than in the unbridged cohort (*P* = .011).

**Conclusions:**

In combination with an azole antifungal, micafungin did not prevent IC in postoperative LTRs with cultures positive for *Candida* species in this cohort. Larger prospective studies are needed to determine the ideal combination and duration of antifungal prophylaxis.

Lung transplantation is increasingly utilized as management for end-stage lung disease, with 1-year survival steadily improving [1]. However, infection continues to be a significant complication, with a 1-year incidence of invasive fungal infections (IFIs) ranging from 10% to 22% [2]. From 1980 to 2004, the incidence of invasive candidiasis (IC) was 5.2%, with infections occurring most commonly within the first 3 months of transplantation [2, 3]. Risk factors for IC in lung transplantation include *Candida* species being ubiquitous and breaches in the epithelial barrier allowing the pathogen to invade the host. Lung transplant candidemia risk factors include pretransplant hospitalization, posttransplant extracorporeal membrane oxygenation (ECMO), and posttransplant renal replacement therapy [4]. The International Society for Heart and Lung Transplantation (ISHLT) recommends antifungal prophylaxis postoperatively [5]. Strategies vary with options including universal prophylaxis administered to all patients after lung transplantation, targeted prophylaxis in patients deemed high risk, or preemptive therapy based on culture or other biomarker results. To date, no prevention strategy has been proven superior to another [6].

Antifungal prophylaxis regimens include nebulized amphotericin and/or systemic triazole (azole) therapy. Nebulized amphotericin is utilized due to its localized effects and low systemic absorption. Azole antifungals have broad-spectrum coverage depending on the agent; however, these agents are limited by their drug–drug interactions with immunosuppressants, the fungistatic mechanism, and the need for therapeutic drug monitoring with the exception of fluconazole and isavuconazole [7]. Conversely, echinocandins demonstrate fungicidal activity against most *Candida* species, are generally well-tolerated with few drug–drug interactions and limited toxicity, and have a favorable pharmacokinetic profile from the first dose.

While echinocandins have proven valuable and efficacious as antifungal prophylaxis in neutropenic patients, their use in lung transplant recipients has not been validated. Furthermore, their use as adjunctive, or “bridge” therapy in the immediate postoperative period during initial azole exposure and discontinued once azole levels are therapeutic has not been studied.

Our center began utilizing micafungin bridging therapy postoperatively in addition to universal azole prophylaxis in recipients with either donor, recipient, or surveillance cultures yielding *Candida* to decrease incidences of IC. This study aimed to determine the effect of micafungin bridging in lung transplantation recipients with positive *Candida* respiratory cultures in the first 6 weeks posttransplantation compared with patients who did not receive micafungin bridging.

## METHODS

This was a single-center, retrospective cohort study conducted via medical record review of lung transplant recipients at the University of Florida Health Shands Hospital between August 2017 and August 2020. Included patients were adult lung transplant recipients with positive *Candida* species respiratory cultures preoperatively (donor or recipient) or within 6 weeks postoperatively by bronchoscopic origin. Donor preoperative respiratory cultures were collected during the final bronchoscopic organ assessment. Sputum cultures were collected preoperatively for recipient patients. In addition, bronchoscopy was performed postoperatively once during days 0–3 to evaluate the anastomosis as well as at weeks 2, 4, and 12, then months 6, 9, and 12. Patients were excluded if they were <18 years old, multiorgan transplant recipients, received ≤72 hours of postoperative micafungin, received micafungin for an indication other than prophylactic “bridge” therapy, or did not have preoperative cultures positive for *Candida* species. Patients were divided into 2 cohorts—unbridged and bridged. This was a pre- and postanalysis as azole monotherapy was utilized prior to 2018 with micafungin bridge adoption in 2019. The unbridged cohort received azole therapy in the immediate postoperative period. The bridged cohort received azole therapy plus micafungin 100 mg intravenously for up to 2 weeks or until azole levels were in the therapeutic range, or at discharge, whichever was sooner. Both cohorts received inhaled amphotericin. We also included patients who did not have surveillance cultures with *Candida* colonization for overall assessment of our patient population (unpublished data). However, as this patient population was not bridged, they were excluded from the analysis herein.

Our institution utilizes a universal antifungal prophylaxis strategy, which includes a risk-based azole alongside nebulized amphotericin B given as 50 mg every 48 hours for 6 doses postoperatively, followed by once-weekly administration during the index admission. Transplant recipients receive either itraconazole 200 mg orally twice daily for 6 months or voriconazole 200 mg orally twice daily for 9 months. Patients with cystic fibrosis, bronchiectasis, or *Aspergillus* history received the latter. Antifungal trough concentrations are drawn at steady-state, with target levels of ≥0.5 μg/mL for itraconazole and ≥1 μg/mL for voriconazole [8]. At transplantation, recipients are administered basiliximab or antithymocyte globulin. Postoperative immunosuppressants include tacrolimus targeting a 10–15 ng/mL trough during the first year, mycophenolate mofetil 1000 mg twice daily, and prednisone initially at 20 mg, tapering to a 5-mg daily maintenance dose. The treating physician assessed acute cellular rejection (ACR), and treatment depended on the pathologic grading. Patients who had mild to severe ACR received methylprednisolone 10 mg/kg daily for 3 days. Treatment options for severe or recurrent acute cellular lung transplant rejection were determined by the treating physician's preference, involving additional therapies such as antithymocyte globulin, photopheresis, and initiation of a mammalian target of rapamycin inhibitor alongside a calcineurin inhibitor. Patients who received methylprednisolone 10 mg/kg daily for 3 days were considered to have rejection and were recorded to have high-dose steroids.

The primary outcome was a composite of proven or probable IC defined by the Revised 2020 European Organization for Research and Treatment of Cancer and the Mycoses Study Group Education and Research Consortium [9]. The Biofire FilmArray Blood Culture Identification Panel (bioMérieux) was employed to identify positive blood cultures, and its results were subsequently confirmed through standard culture. Nonsterile tissue growth of *Candida* species was quantified by light, moderate, or heavy growth. Secondary outcomes included the incidences of proven or probable IFIs, adverse bronchoscopic findings within 6 weeks, sternal wound dehiscence within 6 weeks, bacterial and/or fungal chest wall infections, fungal empyema, intensive care unit (ICU) length of stay, hospital length of stay, and 90-day all-cause mortality. Adverse bronchoscopic findings were defined as anastomotic dehiscence, sloughing, severe ischemia, or severe necrosis documented in the postoperative bronchoscopy report.

Study data were collected and managed using REDCap electronic data capture tools. An a priori significance level was set as *P* < .05. After confirming nonparametric distribution of the data, the Mann-Whitney *U* test was utilized for all continuous data. As appropriate, χ^2^ or Fisher exact test was utilized for categorical data. Analyses were performed on Statistical Package for the Social Sciences (SPSS, Inc, Chicago, Illinois), version 22.0 Premium. This study was approved by the institutional quality improvement program registry (QIPR), which assesses the quality or research nature of a project and grants access to quality improvement data. The QIPR's assessment excluded the need for institutional review board approval in this study.

## RESULTS

A total of 117 patients were included in the study, with 68 in the unbridged cohort and 49 in the bridged cohort. The 2 groups appeared similar with baseline characteristics summarized in Table 1. More patients in the bridged cohort required ECMO and received thymoglobulin or high-dose steroids. Moreover, more patients had their initial antifungal concentration drawn before discharge in the bridged cohort (79.6%) than in the unbridged cohort (39.7%), with 46.9% in the bridged cohort achieving their targeted level. Table 2 shows baseline culture data between the 2 groups. Additionally, it is notable that of the 102 patients throughout the study period who were not colonized with *Candida* species, and thus not bridged, 3 developed IC (data not shown).

**Table 1.** Baseline Characteristics

Characteristic	Unbridged (n = 68)	Bridged (n = 49)
Male sex	34 (50)	25 (51)
Age, y, median (IQR)	63 (54–68)	61 (51–67)
Weight, kg, median (IQR)	69.3 (57.9–84.1)	70.9 (60.3–83.6)
White race	54 (79.4)	35 (71.4)
Lung allocation score, median (IQR)	38.9 (34.1–57.0)	38.5 (33.5–62.0)
Initial transplantation	63 (92.6)	45 (91.8)
Reason for transplantation		
Pulmonary fibrosis	24 (35.3)	22 (44.9)
Cystic fibrosis	3 (4.4)	1 (2.0)
COPD	28 (41.2)	16 (32.7)
Sarcoidosis	2 (2.9)	2 (4.1)
Pulmonary hypertension	0 (0)	1 (2)
Alpha-1 antitrypsin deficiency	2 (2.9)	0 (0)
Bronchiectasis	0	1 (2)
Interstitial lung disease	5 (7.4)	2 (4.1)
Bronchiolitis obliterans	5 (7.4)	4 (8.2)
Required ECMO pre- or posttransplantation	11 (16.2)	16 (32.7)
Transplant type		
Single	5 (7.4)	2 (4.1)
Bilateral	63 (92.6)	47 (95.9)
Induction agent		
Basiliximab	64 (94.1)	46 (93.9)
Received antithymocyte globulin	7 (10.3)	11 (22.4)
Received high-dose steroids postoperatively	8 (11.8)	19 (38.8)
No desensitization history	63 (92.6)	42 (85.7)
History of invasive fungal infection	2 (2.9)	2 (4.1)
Initial azole posttransplant		
Itraconazole	62 (91.2)	45 (91.8)
Voriconazole	6 (8.8)	4 (8.2)
Time to initial azole antifungal, d, median (IQR)		
Itraconazole	1 (1–1)	1 (1–2)
Voriconazole	1 (1–3)	3 (2–4.5)
Initial antifungal level drawn during hospitalization	27 (39.7)	39 (79.6)
Time to initial azole trough TDM, d, median (IQR)		
Itraconazole	13 (10.5–18)	8 (7–15)
Voriconazole	8 (7–9.5)	6 (5–7)
Initial antifungal targeted level achieved	17 (25)	23 (46.9)
Initial antifungal changed during hospitalization	7 (10.3)	11 (22.4)
CMV status		
D^+^/R^–^	21 (30.9)	13 (26.5)
R^+^	33 (48.6)	32 (65.3)
D^–^/R^–^	14 (20.6)	4 (8.2)
Duration of micafungin bridge, d, mean ± SD	…	12 ± 5

Data are presented as No. (%) unless otherwise indicated.

Abbreviations: CMV, cytomegalovirus; COPD, chronic obstructive pulmonary disease; D^–^, donor negative; D^+^, donor positive; ECMO, extracorporeal membrane oxygenation; IQR, interquartile range; R^–^, recipient negative; R^+^, recipient positive; SD, standard deviation; TDM, therapeutic drug monitoring.

**Table 2.** Endpoints

Endpoint	Unbridged (n = 68)	Bridged (n = 49)
Proven or probable IC	0 (0)	5 (10)
Adverse bronchoscopic finding	16 (23.5)	13 (26.5)
Anastomotic wound dehiscence	1 (1.4)	0 (0)
Chest wall infection	2 (2.9)	0 (0)
Sternal wound dehiscence	10 (14.5)	2 (4)
Fungal empyema	0 (0)	3 (6)
ICU LOS, d, median (IQR)	6 (3–9)	9 (5.5–24)
Posttransplant LOS, d, median (IQR)	21 (14–25)	26 (18–41.5)
90-d all-cause mortality	0 (0)	2 (4)

Data are presented as No. (%) unless otherwise indicated.

Abbreviations: IC, invasive candidiasis; ICU, intensive care unit; IQR, interquartile range; LOS, length of stay.

Breakthrough IC was defined as evidence of infection while azoles were therapeutic but not in relation to micafungin. There were no breakthrough IC events with receipt of micafungin bridging. Of the 5 recipients who developed IC, 3 were previously on ECMO and 2 had central venous catheters at diagnosis. One patient who received antithymocyte globulin developed a fungal empyema 10 days later. Of the patients who developed IC, 2 had breakthrough fungal empyema despite therapeutic itraconazole concentrations. One patient with IC empyema had a subtherapeutic itraconazole level. The remaining 2 patients did not have concentrations recorded to evaluate for subtherapeutic azole concentrations. Moreover, all patients who developed fungal empyemas had indwelling chest tubes.

### Primary Outcome

There were more cases of IC in the bridged cohort compared to the unbridged cohort (*P* = .01) (Table 3). Most infections were isolated from the pleural fluid (4 of 5). *Candida albicans* caused 60% of the IC cases (Table 3). The median time to IC occurrence was 18 days.

**Table 3.** Invasive Candidiasis Characteristics

Characteristic	Bridged (n = 5)	
Blood		
*Candida albicans*	1	
Pleural fluid		
*Candida albicans/dubliniensis*	2	
*Candida glabrata*	1	
*Candida guilliermondii*	1	

### Secondary Outcomes

There were no incidences of IFIs in this cohort, only IC. There was no difference in adverse bronchoscopic events between the 2 groups (*P* = .7). One recipient in the unbridged cohort had evidence of anastomotic wound dehiscence. No recipients in the unbridged group developed a fungal chest wall infection. There was no difference in anastomotic wound dehiscence between the 2 groups (*P* = .1). Patients received a mean micafungin duration of 12 days.

## DISCUSSION

The ISHLT recommends antifungal prophylaxis; however, there is no consensus on the optimal approach with options ranging from universal to preemptive. Furthermore, current guidelines recommend antifungal therapy if donor bronchopulmonary secretions yield *Candida* until anastomosis evaluation [10]. This study aimed to address if using targeted micafungin bridging in lung transplant recipients who had positive perioperative or surveillance cultures would decrease the incidence of IC. Combined with inhaled amphotericin B and azole therapy, bridging with micafungin until therapeutic azole concentrations achieved did not reduce the incidence of IC.

Risk factors for IC include prolonged use of broad-spectrum antibiotics, presence of a central venous catheter, and need for renal replacement therapy; however, this was not evaluated in this study [11]. A more recent analysis reported the epidemiology and outcomes of IC in solid organ transplant recipients and reported that 8.7% of lung transplant recipients develop IC, with a 36.4% mortality rate [12]. The median time to onset for IC was 80 days posttransplantation. Moreover, bloodstream and pulmonary site infections occurred in 44.8% and 5.6% of lung transplant recipients, respectively. In our study, we identified more incidences of thoracic infections. Our IC findings are similar to Linder et al, with 4 proven *Candida* infections involving the thoracic structure despite lung transplant recipients receiving targeted prophylaxis [13]. In their cohort, 70% of recipients developed proven/probable invasive aspergillosis. In our study, there were no incidences of proven or probable invasive pulmonary aspergillosis; however, this may be attributed to the restrictive timeframe of 6 weeks.

A worldwide survey for antifungal prophylaxis in lung transplant recipients found that of the 34 centers that responded, only 11 used combination therapy with azole and inhaled amphotericin B [14]. In the survey, only 1 center used combination therapy with voriconazole and micafungin. In a United States survey, echinocandins were used in 2 of 34 centers in the immediate postoperative period [15]. These surveys demonstrate the institutional variations in preventing IFI and the limited studies evaluating IFI outcomes based on institutional protocols.

Baker et al retrospectively evaluated their antifungal prophylaxis protocol from January 2007 to October 2014 [16]. Their protocol used nebulized amphotericin B and targeted systemic prophylaxis with a mold-active azole. Patients who had delayed chest closure after transplantation or required postoperative ECMO received micafungin prophylaxis until chest closure or ECMO decannulation. Proven or probable IFIs were analyzed during the first 180 days after lung transplantation. The median time to an IC event was 31 days. One hundred fifty-six of 815 patients developed an IFI. Among those patients, 96 episodes of IC were recorded, and 18% of IC events occurred despite prophylactic therapy, with 89% receiving micafungin prophylaxis. Azole therapeutic drug monitoring was not utilized in this study. The authors also identified local risk factors that contribute to IFIs, including postoperative ECMO use, high lung allocation score, graft retransplantation, prolonged postoperative mechanical ventilation, and bilateral lung transplantation.

Our study found that donor airway *Candida* colonization may not be associated with contributing to IC. Only 1 patient developed IC with *Candida glabrata* that may have been donor-derived, whereas the other patients had differing *Candida* species compared to preoperative cultures (Supplementary Table 1). The absence of a clear correlation between donor culture *Candida* species and the subsequent infections observed in our study may be attributed to limitations in *Candida* speciation methods. Matrix-assisted laser desorption/ionization–time of flight may not reliably distinguish closely related species like *Candida albicans* and *Candida dubliniensis*. Additionally, the dynamic nature of the lung microenvironment during transplantation, including the effects of surgical procedures and changes in local immunity, could alter the composition of the lung flora, potentially impacting *Candida* colonization. Further research, utilizing more precise speciation techniques and considering the intricate factors at play, is warranted to elucidate this complex phenomenon in lung transplant recipients. Despite *Candida* species frequently being considered a colonizer, there have been cases reporting donor-derived infections in lung transplant recipients [17, 18]. Administration of 2 weeks of micafungin for esophageal candidiasis in immunocompromised patients found that eradication rates ranged between 32% and 78% [19]. In our study, we observed that even after administering therapeutic triazole antifungal treatment and 2 weeks of micafungin, 8% of our patients continued to exhibit colonization by *Candida.*

Our findings share similar results with Sartain et al [20]. Their retrospective study evaluated a pre- and postprotocol implementation of anidulafungin with itraconazole, until serum concentrations were ≥0.5 μg/mL or hospital discharge, and its effect on IC. Of note, the median itraconazole concentration was 0.075 mg/mL in the preprotocol cohort and 0.095 mg/mL in the protocol cohort. There was no statistically significant difference in proven or probable IC between the 2 groups (*P* = .16); however, there was an overall decreasing trend in *Candida* species causing IFIs. In their multivariate analysis, risk factors that increased the incidence of proven or probable IFIs included transplant length of stay, lung allocation score (which declined from 38.2 in the preprotocol cohort to 34.5 in the protocol cohort), perioperative donor culture with fungal growth, bilateral lung transplantation, and anastomotic or sternal dehiscence. In our study, higher lung allocation scores, donor *Candida* colonization, and anastomotic/sternal dehiscence did not contribute to an increased risk of developing IFIs. Moreover, patients in our cohort who were bridged with micafungin had a longer ICU length of stay and indwelling chest tubes. This criterion has been associated with IC [21].

Echinocandin therapy risks should be carefully considered before broad adoption in large patient populations. Echinocandins may exhibit a selective pressure leading to either echinocandin tolerance or overt echinocandin resistance [22, 23]. Global trends have displayed decreased *C albicans* isolation and increased *C glabrata* isolation, both with increasing echinocandin resistance [24, 25]. A recent 10-year survey at a large tertiary transplant center reported increased echinocandin resistance from 4.9% to 12.3% [26]. Prior echinocandin exposure was associated with an increased risk (odds ratio, 5.3) of developing nonsusceptible *C glabrata* infections [27]. Furthermore, micafungin has poor penetration into the pleural cavity, the majority of our IC being isolated in pleural fluid. Last, micafungin is extracted by the ECMO circuit with 32.7% of the bridge cohort requiring ECMO either pre- or posttransplantation [16].

Deviations from our institutional protocol included the following: no azole antifungal prophylaxis 24 hours after lung transplant surgery and delayed therapy in select patients without documentation. In addition, some patients had overlap with micafungin despite having therapeutic azole levels or prolonged duration of micafungin by >14 days. These deviations were due to the treating physician's preference, as these patients were perceived as more critically ill with either longer ECMO duration or highly immunosuppressed after desensitization. Although these deviations occurred, they did not impact study endpoints. Patients on ECMO therapy at our institution received 100 mg of micafungin and were not dose adjusted despite conflicting evidence that higher doses may achieve more optimal pharmacokinetic exposure [28, 29].

This study had several limitations, such as increased susceptibility to a type II error given the retrospective, single-center design and small sample size. The lack of evaluation of donor, recipient, or surveillance culture-negative patients for *Candida* species at baseline and extending beyond 6 weeks posttransplantation may have limited the detection of IC, epidemiology, and adverse events beyond that time. A limitation of our study is the lack of quantifying data on fungal growth in donor or recipient lungs. This absence of quantitative information makes it challenging to assess the potential relationship between higher quantities of *Candida* organisms in donor bronchoscopic cultures at the time of transplantation and the predisposition of patients to pleural IC, possibly due to contamination of the pleural cavity during transplantation.

We did not evaluate other known risk factors for IC, such as central lines, renal replacement therapy, diabetes mellitus, and broad-spectrum antimicrobial administration. There is also the possibility of antifungal extraction by ECMO in which micafungin doses were kept at 100 mg; however, this was less likely to affect study outcomes as patients developed IC after ECMO discontinuation. As mentioned previously, 2 patients who had IC did not have azole concentrations collected, which may have contributed to IC. We also did not stratify patients where bridging may be appropriate and did not address if patients had severe ACR requiring other therapeutic modalities. Due to the small sample size, a multivariate analysis for identifying factors contributing to IC was not conducted to avoid issues like overfitting and unreliable estimates. The necessity for a rigorous assessment of *Candida* colonization in the context of lung transplantation warrants a case-specific approach. The study could not address emerging fungal resistance within the prespecified timeframe despite the incidence of increased fungal resistance from prior studies. An extended study duration may provide institutional data for developing resistance. Since only *Candida* species were the only documented IFI, this study should not be extrapolated for adjunctive agents in the postoperative period for other fungi.

## CONCLUSIONS

In combination with an azole antifungal, micafungin did not prevent IC in postoperative lung transplant recipients with donor or recipient respiratory cultures positive for *Candida* species. A prospective, randomized, controlled trial assessing noninferiority with echinocandin bridging and azole prophylaxis monotherapy is warranted.

## Supplementary Data

Supplementary materials are available at *Open Forum Infectious Diseases* online. Consisting of data provided by the authors to benefit the reader, the posted materials are not copyedited and are the sole responsibility of the authors, so questions or comments should be addressed to the corresponding author.

## Supplementary Material

ofae525_Supplementary_Data

## Notes

***Patient consent.*** This manuscript does not contain elements requiring patient consent.
