# Supplementary material for: Bridging Echinocandin With Azole Antifungal Therapy on Prevention of Invasive Candidiasis Post–Lung Transplantation
Source: Open Forum Infect Dis. 2024 Sep 11;11(9):ofae525. doi: 10.1093/ofid/ofae525 (PMC11425485; doi:10.1093/ofid/ofae525)
Supplement: ofae525_Supplementary_Data [file ofae525_supplementary_data.docx]

OFID Supplementary

**Supplemental Table 1: Bronchoalveolar lavage *Candida* culture data pre- and postoperatively**

|  | Unbridged  (n=68) | Bridged  (n=49) |
| --- | --- | --- |
| Donor | 30 (44.1) | 43 (87.8) |
| *Candida albicans* | 28 | 37 |
| *Candida dublinensis* | 27 | 33 |
| *Candida glabrata* | 3 | 6 |
| *Candida krusei* | 0 | 1 |
| *Candida tropicalis* | 1 | 5 |
| *Candida parapsilosis* | 1 | 6 |
| Recipient | 4 (5.9) | 4 (8.2) |
| *Candida albicans* | 3 | 2 |
| *Candida dublinensis* | 2 | 3 |
| *Candida glabrata* | 0 | 2 |
| *Candida tropicalis* | 1 | 0 |
| Surveillance culture within 6 weeks | 35 (51.5) | 4 (8.2) |
| *Candida albicans* | 29 | 4 |
| *Candida dublinensis* | 26 | 4 |
